# Increasing Seaweed Consumption in the Netherlands and Portugal and the Consequences for the Intake of Iodine, Sodium, and Exposure to Chemical Contaminants: A Risk-Benefit Study

**DOI:** 10.3389/fnut.2021.792923

**Published:** 2022-01-06

**Authors:** Reina Elisabeth Vellinga, Matthijs Sam, Hans Verhagen, Lea Sletting Jakobsen, Gitte Ravn-Haren, Minami Sugimoto, Duarte Torres, Ryoko Katagiri, Beate Julie Thu, Kit Granby, Jeljer Hoekstra, Elisabeth Helena Maria Temme

**Affiliations:** ^1^Centre for Nutrition, Prevention and Health Services, National Institute for Public Health and the Environment (RIVM), Bilthoven, Netherlands; ^2^National Food Institute, Technical University of Denmark, Lyngby, Denmark; ^3^Department of Social and Preventive Epidemiology, Division of Health Sciences and Nursing, Graduate School of Medicine, The University of Tokyo, Tokyo, Japan; ^4^Epidemiology Research Unit, Institute of Public Health, University of Porto, Porto, Portugal; ^5^Laboratory for Integrative and Translational Research in Population Health (ITR), Porto, Portugal; ^6^Faculty of Nutrition and Food Sciences, University of Porto, Porto, Portugal; ^7^Division of Epidemiology, Center for Public Health Science, National Cancer Center, Tokyo, Japan; ^8^Moreforsking, Marine Ecosystems, Alesund, Norway

**Keywords:** seaweed, risk-benefit, sodium, iodine, arsenic, cadmium, lead, mercury

## Abstract

**Background:** Seaweed has a high potential for nourishing the future planet. However, besides being beneficial, it also contains adverse components; this poses the question whether consumption of seaweed foods overall contributes beneficially or detrimentally to human health, and hence if their consumption should be promoted or restricted.

**Methods:** This study evaluated the impact of substituting regular foods with seaweed foods in the diet, both in terms of nutritional quality (*via* iodine and sodium) and food safety (*via* arsenic, cadmium, lead, and mercury). Food consumption data from the Netherlands and Portugal (adults aged >18 years) were used, in which 10% of the amounts of pasta, bacon, and lettuce consumed were replaced by seaweed-derived products made from kelp (*Saccharina latissima*). Using Monte Carlo Risk Assessment software (MCRA), long-term nutrient intake and exposure to contaminants were assessed. The results obtained for the Netherlands and Portugal were compared with data from Japan, a country that has a high natural consumption of seaweed.

**Results:** This low-tier risk-benefit study reveals that an increased seaweed consumption (as assessed by the 10% replacement with seaweed products) has no consequences in terms of intake of sodium and exposure to cadmium, lead, and mercury, and the associated (absence of) adverse health aspects. The alternative scenario almost doubled the mean iodine intake in the Netherlands (to 300 μg/day) and Portugal (to 208 μg/day) and increased the average exposure to arsenic levels in the Netherlands (to 1.02 μg/kg bw/day) and Portugal (to 1.67 μg/kg bw/day).

**Conclusion:** The intake of iodine and exposure to arsenic in the Netherland and Portugal were certainly higher due to the modeled increase of seaweed foods. If seaweed consumption increases close to the 10% substitution, the public health consequences thereof may trigger further research.

## Introduction

Considering the growing world population and environmental challenges we are facing, changes in food production and food consumption are required. Aquaculture and fisheries can be a key to the future of food production and nutritious food systems and should address these challenges (1). The consortium SEAFOODTOMORROW aims to develop new environment-friendly and transparent seafood production and processing methods that will improve European seafood security (https://seafoodtomorrow.eu/). Seaweed is one of the several sustainable opportunities among seafood that a has high potential for nourishing the future planet (2).

The interest in developing an industrialized cultivation technique for seaweed in Europe is growing rapidly, as seaweed has raised public awareness because of several reasons. Seaweed grows easily on large scale in the sea, has several applications (e.g., as fuel, feed, or as food) and is seen as an alternative food with great potential (3, 4). The composition of seaweed varies between species, but in general seaweed is low in fat and includes a range of essential and beneficial nutrients (5), such as omega *n*-3 fatty acids, vitamins (A, C, and E), iodine, dietary fiber, and antioxidants (6–8). Seaweed as (novel) food for human consumption has increased over the past years (e.g., in the UK), but the current consumption in Western countries is low and seaweed usually is not part of the diet (6, 9, 10).

Seaweed can contain high concentrations of iodine and can be considered as “rich in” iodine according to Regulation EU 1924/2006 (11, 12). Globally, the elimination of iodine deficiency is regarded as a major public health challenge (13–16). Iodine deficiency results in inadequate thyroid hormone production causing a range of adverse health effects, including impaired growth and development in children and in the offspring of deficient mothers, cretinism, goiter, and thyroid cancer (17). Previous studies investigating iodine status in Portuguese pregnant women and school-aged children reported intakes well below the adequacy recommendation of WHO (13, 14). Verkaik-Kloosterman et al. reported that the iodine intake among the Dutch population (7–69 years) seems adequate due to fortification of bread with iodized salt, although iodine intake has decreased since the period before 2008 (16). Contrary, high iodine intake exceeding the upper tolerable intake levels may lead to iodine toxicity and can have negative health effects such as impaired thyroid function, goiter, and hyperthyroidism (9, 18). Moreover, seaweed contains the essential micronutrient sodium. The intake of sodium is, in general, much higher than recommended and should be limited. High levels of sodium intake are associated with adverse health outcomes, such as (elevated) blood pressure, cardiovascular disease-related endpoints, and bone health (19).

Whereas seaweed contains a number of beneficial nutrients, seaweed may also accumulate and be a dietary source of exposure to heavy metals such as arsenic (As), cadmium (Cd), lead (Pb), and mercury (Hg) (8, 20, 21). Long-term dietary exposure to these compounds or their metabolites may lead to a wide range of adverse health effects. Inorganic arsenic (i-arsenic) is classified as a human carcinogen and causes cancer to the lung, bladder, and skin (22); cadmium causes chronic kidney disease (23), and methyl-mercury (24) and lead (25) are associated with impaired neurodevelopment in fetuses and young children and cardiovascular disease. For most countries, there is currently no regulation on the maximum levels of the above mentioned chemical contaminants in seaweed, apart from cadmium concentrations in France (0.5 mg/kg as a maximum level for cadmium in dried seaweed) (26). Additionally, although the EU has no regulation on the maximum level of iodine in algae food products, France has set a limit of 5 mg/kg dry matter and Germany warns about damage to health for algae food products with more than 20 mg/kg dry matter algae (27).

As seaweeds contain both nutrients and contaminants, it poses the question whether consumption of these foods has a beneficial or detrimental effect on human health and whether consumption should be promoted or restricted. This is the domain of risk-benefit assessment in food safety and nutrition, an emerging field in food safety risk assessment (28–32). In this study, we evaluated the impact of replacing regular pasta, bacon, and lettuce with similar foods produced from seaweed in the diet. The problem formulation in this study is: ”What is the impact of exchanging 10% of pasta, bacon, and lettuce with similar foods produced from seaweed in terms of total intake and exposure of adults in the Netherlands and Portugal to selected nutrients (sodium and iodine) and contaminants (arsenic, cadmium, lead, and mercury)?” Two European countries (the Netherlands and Portugal) are included, which represent European diets. With a low tier risk-benefit analysis, we examine the change in intake of selected dietary nutrients (iodine and sodium) and chemical contaminants [total-arsenic (organic and inorganic arsenic) (t-arsenic), i-arsenic, cadmium, lead, and mercury]. A rough comparison is made with established health-based guidance values (HBGVs) or benchmark dose lower confidence limit (BMDLs) by EFSA or FAO/WHO Joint Expert Committee on Food Additives (JECFA). The results obtained for the Netherlands and Portugal are compared with dietary intake data from Japan, a country with a high natural consumption of seaweed.

## Materials and Methods

### Alternative Scenario

To estimate the intake and exposure of seaweed products for the Netherlands and Portugal, a scenario derived from the current consumption patterns was developed. In this alternative scenario, 10% of the consumption of pasta, bacon, and lettuce was replaced by seaweed pasta, seaweed bacon, and seaweed lettuce, respectively. Those seaweed products are currently on the market in the Netherlands. The seaweed products were entirely made from kelp (*Saccharina latissima)*. In the Dutch and Portuguese populations, the consumption of pasta, bacon, and lettuce in grams per person per day was identified and 10% of the amounts were replaced by seaweed pasta, seaweed bacon, and seaweed lettuce (Table 1).

**Table 1:** Scenarios of replacement of foods by seaweed-derived products.

**Reference scenario**	**Alternative scenario**
Pasta^a^	10% of the habitual consumption of pasta per person per day is replaced by seaweed pasta
Bacon^b^	10% of the habitual consumption of bacon per person per day is replaced by seaweed bacon
Rocket lettuce^c^	10% of the habitual consumption of rocket lettuce per person per day is replaced by seaweed lettuce

a *Foodex2 codes A007D A007E A04LC A007F A007G A007J A007L A007M A007P*.

b *Foodex2 code A022X*.

c *Foodex2 code A00LN*.

### Food Consumption Data From National Food Consumption Surveys

For the Netherlands, representative consumption data of 2,078 participants aged 19–79 years were derived from the Dutch National Food Consumption Survey (DNFCS) 2012–2016 (33). Food consumption was assessed by two nonconsecutive 24-h dietary recalls approximately four weeks apart, and the interviewers used the GloboDiet system (34). This is a computer-controlled interview software with which answers are directly entered onto a computer by a trained dietician. The reported foods and recipes in the DNFCS 2012-2016 were described according to the GloboDiet methodology. Foods were labeled with NEVO food codes according to the Dutch Food Composition Database (NEVO) (2016/5) (35) and to the FoodEx2 classification system (36). The use of supplements and added (iodized) table salt was not included in the current analysis.

For Portugal, representative consumption data were derived from the National Food and Physical Activity Survey (IAN-AF 2015-2016), including 3,792 Portuguese participants aged 19–84 years (37). Food consumption was assessed by two nonconsecutive 24-h dietary recalls conducted 8–15 days apart, using a validated computer-assisted tool (eAT24)(38). Foods were described according to the EFSA FoodEx2 classification system (36). The use of supplements and added (iodized) table salt was not included in the current analysis.

For Japan, consumption data were taken from a survey conducted in four geographically separated areas of Japan, namely Osaka (Osaka City, urban area), Okinawa (Ginowan City, urban island area), Nagano (Matsumoto City, rural inland area), and Tottori (Kurayoshi City, rural coastal area) (39, 40). The survey included 120 women aged 30–69 years and their husbands (240 participants in total). Food consumption was assessed by four semiweighted food records on nonconsecutive days, in each of the four seasons, resulting in sixteen measurement days per participant. Foods were described according to the EFSA FoodEx2 classification system (36). Foods that were only consumed by the Japanese population were matched to FoodEx2 codes of comparable European foods based on the ingredient composition. For example, Onigiri (Japanese rice ball) was matched to the FoodEx2 code for “Rice and similar” or traditional Japanese confectionary such as “Uguisu-mochi” (rice cake filled with red bean paste) was matched to the FoodEx code for “Sugar and similar, confectionery and water-based sweet desserts.” Japanese data was intended for comparison with study outcomes for the Netherlands and Portugal. The use of supplements was excluded, whereas added table salt was included in the current analysis.

### Food Composition Data for Iodine and Sodium

For the Netherlands, iodine and sodium concentrations were derived from the Dutch Food Composition Database (NEVO) (2016/5) (35) and matched with food consumption data using NEVO food codes.

For Portugal, the Portuguese Food Composition Table (PFCT) provided sodium concentrations (41). Iodine concentrations were derived from alternative sources, namely the EFSA occurrence database (measured between 2014 and 2018) [Food composition | European Food Safety Authority (europa.eu)] and WHO (42). FoodEx2 codes were used to match foods between PFCT and EFSA or WHO databases. The remaining foods for which no concentrations were available from EFSA or WHO were matched to iodine concentrations from NEVO (2016/5) (35). NEVO food codes were linked to foods from the Portuguese food consumption survey using the FoodEx2 classification system. In case no corresponding FoodEx2 classification code was available, foods were ascribed an average aggregated concentration based on corresponding FoodEx2 hierarchy.

For Japan, iodine concentrations were derived from a comprehensive iodine content database (39), which was developed based on the Standard Table of Food Composition in Japan and analytical values from other works. The Standard Table of Food Composition in Japan provided sodium concentrations (43). Concentration data were previously linked to consumption data.

An overview of sodium and iodine concentrations used for replacement foods in the alternative scenario is provided in Table 2. Iodine and sodium concentrations in seaweed pasta and seaweed bacon (*Saccharina latissimi)* were derived from the back-of-pack nutritional information of seaweed food products available in the Dutch market (44, 45). Iodine and sodium concentrations for seaweed lettuce (‘Seaweed kelp raw’) were derived from the Dutch Food Composition Database (NEVO 2016/V5) (35). In NEVO, the concentrations for iodine (46) and sodium (47) in seaweed were both derived from the food composition table of Denmark and the US, respectively.

**Table 2:** Concentrations of iodine, sodium, arsenic, cadmium, lead, and mercury in unit per kg of pasta, bacon, lettuce, and novel seaweed foods.

	**Pasta**	**Seaweed pasta (WW)**	**Bacon**	**Seaweed bacon (DW)**	**Lettuce**	**Seaweed lettuce (WW)**
**Nutrients**						
Iodine (μg/kg)	NL: 10^a^	29200^c^	NL:136^a^	163000^c^	0^a^	75530^a^
	PT: 9^a^		PT:112^a^			
Sodium (mg/kg)	NL: 55^a^	7240^c^	NL:12452^a^	12400 ^c^	250^a^	2330^a^
	PT: 50^a^		PT:15400^a^			
**Heavy metals**						
t-Arsenic (μg/kg)	21^b^	8582^d^^,^^e^	15^b^	40333^d^	20^b^	8582^d^^,^^e^
i-Arsenic (μg/kg)	15^b^	11^e^^,^^f^	11^b^	50^f^	13^b^	11^e^^,^^f^
Cadmium (μg/kg)	14^b^	94^d^^,^^e^	7^b^	443^d^	36^b^	94^d^^,^^e^
Lead (μg/kg)	8^b^	24^d^^,^^e^	11^b^	111^d^	30^b^	24^d^^,^^e^
Mercury (μg/kg)	10^b^	< LOQ (0.90)^d^^,^^e^	3^b^	< LOQ (0.90) ^d^	2^b^	< LOQ (0.90)^d^^,^^e^

a *Mean concentrations NEVO or PFCT for iodine and sodium*.

b *Mean concentrations EFSA for heavy metals*.

c *Source: seaweed pasta (“I Sea Pasta”); seaweed bacon (“I Sea Bacon”)*.

d *Mean concentrations IMTA macroalgea (Seafood Tomorrow data)*.

e *Concentrations were divided by a conversion factor of 4.7 for converting dry weight (DW) to wet weight (WW)*.

f *Mean concentration of heavy metals in Saccharina latissimi measured by The Norwegian National Institute of Nutrition and Seafood Research (NIFES)*.

*NL, Netherlands; PT, Portugal*.

### Content of Chemical Contaminants in Food

For the Netherlands, country-specific concentration data on t-arsenic, i-arsenic, cadmium, lead, total mercury (t-mercury), and methyl-mercury from 2014–2017 (48) were derived from the Quality of Agricultural Products (KAP) database, which contains data on contaminants, pesticides, and residues in food and animal feed. KAP database stores data from measurements of residues of pesticides, veterinary medicines, and contaminants from various Dutch monitoring programs provided by the Dutch Food and Consumer Product Safety Authority (NVWA) and Wageningen Food Safety Research (WFSR). The NVWA inspects only a selected range of foods each year, and so to cover the entire diet, concentration data were also derived from the European Food and Safety Authority (EFSA) reports on dietary exposure to heavy metals (24, 25, 49–51). These reports include the mean concentration of on t-arsenic, i-arsenic, cadmium, lead, t-mercury, and methyl-mercury in foods reported to the EFSA by European countries (24, 25, 49–51). The FoodEx2 hierarchy was used to ascribe an average concentration per food. For lead and i-arsenic occurrence, data were reported on hierarchy level 3 (e.g., “Beef,” “Pork,” “Goat,” “Chicken,” etc.), for t-arsenic and cadmium data were reported on hierarchy level 2 (e.g. “Bread and rolls,” “Pasta,” “Fine bakery wares,” etc.), and for t-mercury data was reported on hierarchy level 1 (e.g., “Meat and meat products,” “Fish and other seafood,” “Fruit and fruit products,” etc.).

For Portugal, country-specific concentration data on cadmium and t-mercury were derived from the EFSA occurrence database of foods analyzed between 2014 and 2018 (https://www.efsa.europa.eu/en/call/call-continuous-collection-chemical-contaminants-occurrence-data-0, https://www.efsa.europa.eu/en/data/food-composition). Concentration data on t-arsenic, i-arsenic, and lead were derived from the EFSA reports on dietary exposure to heavy metals (24, 25, 49–51). The FoodEx2 hierarchy was used to ascribe an average concentration for i-arsenic, t-arsenic, cadmium, lead, and t-mercury per food. Furthermore, if for a specific food no EFSA concentration data were available, Dutch country-specific concentration data analyzed between 2014 and 2017 from the KAP database was used.

For Japan, country-specific concentration data on t-arsenic, i-arsenic, cadmium, lead, t-mercury, and methyl-mercury were not available. Therefore, Japanese food consumption data were linked to the EFSA concentration data reported in the EFSA reports on dietary exposure to heavy metals (24, 25, 49–51). Japanese foods and dishes were linked to corresponding FoodEx2 codes. The FoodEx2 hierarchy was used to ascribe an average concentration for t-arsenic, i-arsenic, cadmium, lead, and mercury per food. In Japan (also) other species of seaweed were consumed than those available in the EFSA databases. Therefore, with this method only a rough estimate of Japanese exposure can be given.

For replacement foods in the alternative scenario, levels of t-arsenic, i-arsenic, cadmium, lead, and mercury in seaweed pasta, seaweed bacon, and seaweed lettuce were derived from mean concentrations reported by the SEAFOODTOMORROW consortium (specific data has not been published yet). These concentrations were comparable with the concentrations reported in literature based on measurements from *S. latissima* cultivated in Norwegian waters (52, 53). For concentrations in seaweed lettuce and pasta, a conversion factor of 4.7 was used to convert the dry weight concentrations to wet weight concentrations for t-arsenic, i-arsenic, cadmium, lead and mercury. It was assumed that seaweed lettuce and bacon is consumed fresh (wet weight). The conversion factor was based on food composition of wet and dry seaweed that were included in the Dutch Food Composition Database (35).

Table 2 gives an overview of concentrations of iodine, sodium, lead, t-arsenic, i-arsenic, cadmium, lead, and mercury in unit per kg of regular pasta, bacon, lettuce, and the replacement foods.

### Guidance Values

The resulting usual daily intake and exposure for iodine, sodium, and chemical contaminants in the Netherlands, Portugal, and Japan were compared with the most recent evaluations of HBGV's, as published by EFSA and JECFA (FAO/WHO Joint Expert Committee on Food Additives). For sodium and iodine, HBGV's, which are the dietary references values, are set in mg/day and μg/day, respectively. For cadmium and mercury, the HBGVs are set by EFSA/JECFA in provisional tolerable weekly intake [(P)TWI] or provisional tolerable monthly intake [(P)TMI] levels, resulting from their long half lives in the human body; these (P)TWI or (P)TMI levels were recalculated into daily exposure (μg/kg bw/day) to allow for comparison.

For iodine, an adequate intake (AI) for adults was set by EFSA at 150 μg/day (54), and a tolerable upper intake level (UL) at 600 μg/day (18). The AI was set based on prevalence of thyroid volume enlargement (goiter) in school-aged children, showing a prevalence of goiter below 5% at urinary iodine concentrations above 100 μg/L. This threshold for sufficient iodine intake in children is proposed to be applied for adults and corresponds to an approximate iodine intake of 150 μg/day. Taking into account an increased iodine demand for thyroid hormone synthesis and fetus development during pregnancy, an AI of 200 μg/day is proposed by EFSA for pregnant and lactating women, provided their iodine stores are adequate before pregnancy. The UL was established based on studies reporting marginal biochemical changes in thyroid-stimulating hormone (TSH) levels and in TSH response to thyrotropin-releasing hormone stimulation after iodine intake. The biochemical changes were not associated with any clinical adverse effect at the reported intakes of 1,700 and 1,800 μg/day. An uncertainty factor of three was applied in deriving the UL of 600 μg/day for adults including pregnant and lactating women.

For sodium, available data are not sufficient to establish an AI or UL, and a daily intake of 2,000 mg/day is set as a “safe and adequate intake” for the general adult European population, including pregnant and lactating women (19). The evidence for a relationship between increased sodium intake and elevated blood pressure is strong, and the proposed intake level takes into account both maintenance of sodium balance and the reduced risk of hypertension and cardiovascular disease.

For cadmium, EFSA in 2009 did set a (P)TWI at 2.5 μg/kg bw/week based on toxicity to the proximal tubular cells of the kidney as critical effect, which is recalculated for the purpose of this study into 0.36 μg/kg bw/day (23). In 2013, JECFA did set a (P)TMI at 25 μg/kg bw/month, which is recalculated for the purpose of this study into 0.83 μg/kg bw/day (55). As the EFSA (P)TWI value is lower, *viz* more conservative, the EFSA (P)TWI value will be used for comparing exposure data rather than the JECFA (P)TMI, even though the JECFA HBGV is slightly more recent.

For mercury, a distinction can be made between inorganic mercury and methyl-mercury. For methyl-mercury, JECFA in 2007 set a (P)TWI at 1.6 μg/kg bw/week, whereas EFSA in 2015 set a (P)TWI at 1.3 μg/kg bw/week based on neurodevelopment toxicity as critical effect and expressed as mercury (56). Since both values are close to one another, only the most recent and most conservative value will be considered for the purpose of this study, and that week-value is recalculated into 0.19 μg/kg bw/day. For inorganic mercury, both EFSA (2015) and JECFA (2011) did set a (P)TWI at 4 μg/kg bw/week based on nephrotoxicity as the critical effect, which is recalculated, for the purpose of this work, into 0.57 μg/kg bw/day.

For arsenic and lead, exposure was compared with EFSA and/or JECFA derived from the lower confidence limit of the calculated benchmark doses (BMDL), which are to be used as reference points in margin of exposure (MoE) evaluations. This is because the relevant critical toxic effects are considered non-threshold in nature and thus exposure level can be considered from the perspective of high or low health concern.

For arsenic, a distinction is made between t-arsenic and i-arsenic. Organic arsenic is the main occurring form in fish and seafood (57), and is widely assumed to be of no toxicological concern (50). Consequently, EFSA and JECFA have derived reference doses for i-arsenic specifically. As reference points for i-arsenic, JECFA identified a benchmark dose lower confidence limit of 3.0 μg/kg bw/day for a 0.5% increased incidence (BMDL0.5) of lung cancer (58). EFSA identified a benchmark dose lower confidence limit for a 1% increased incidence (BMDL01) for risk of cancer of the lung, skin, bladder, and also skin lesions between 0.3 and 8 μg/kg bw/day (49). Exposure to both t-arsenic was compared with the EFSA reference dose for i-arsenic; thus a conservative approach was used for t-arsenic.

For lead, EFSA (2012) identified age-dependent reference doses, of which the following two are relevant for men and women from 18 years of age: 1.5 μg/kg bw/day, based on a BMDL01 for a 1% increase in systolic blood pressure as critical effect, and 0.63 μg/kg bw/day based on a BMDL10 for a 10% increase in incidence for chronic kidney disease as critical effect (59). Since this report focusses solely on exposure in adults, these two reference doses are both considered applicable.

### Data Analysis

All analyses were separately executed for the Netherlands, Portugal, and Japan. Descriptive statistics for population characteristics and average consumption of foods (i.e., seaweed) for the total population and for consumers only among the three countries were estimated using SAS software, version 9.4 (SAS Institute Inc., Cary, NC, USA) and reported as mean ± standard deviation (std), percentile distributions or number (*n*)/percentage (%).

Dietary iodine and sodium intake and exposure to t-arsenic, i-arsenic, cadmium, lead, and mercury for the reference (all three countries) and alternative scenarios (for the Netherlands and Portugal only) were assessed using the statistical software Monte Carlo Risk Assessment (MCRA), version 8.3 (60). To estimate the exposure to contaminants, when the concentrations in the food samples were below the limit of detection (LOD) or quantification (LOQ), concentrations equal to ½ LOD/LOQ were assigned. A logistic normal normal (LNN) model was applied to estimate the chronic long-term intake and exposure upon assessment, if positive intake and exposure distributions were normally distributed (61). For the daily exposure distribution, a logarithmic transformation was used, and the correlation between intake frequency and amount was assumed zero. Cooked amounts of foods were used to estimate the intake of nutrients and raw amounts were used to determine chemical contaminant exposure. Estimates for the Dutch population were weighted for small differences in demographic properties, season, and combination of both consumption days (week or weekend) to make results representative for the Dutch population. For Portugal sampling weights were applied to overcome the different probability of sampling units selection; the different probability of individuals selection in each unit, by sex and age (considering the total population, by sex and age groups in the closest recruitment wave); and to correct for bias. For analyses considering Japanese data, no sampling weights were applied. Non-response estimated nutrient intakes were expressed as μg/day or mg/day and reported as mean with 95% confidence interval (CI), 50th, 75th, and the 95th percentile with 95% CI. The long-term exposure distribution for chemical contaminant was expressed as μg/kg body weight (bw)/day and reported percentiles included the mean, 50th, 75th, and 95th percentile with 95% CI. Descriptive statistics and 95% CI were used to determine differences in intake or exposure for the alternative scenario compared with the reference scenario.

## Results

The average (mean ± SD) age of the Dutch population (*n* = 2,078; 50% women) was 50 ± 19 years and the average (mean ± SD) BMI was 26.3 ± 5.1 kg/m^2^ (Table 3). The Portuguese population (*n* = 3,792) included 53% women and was on average 48 ± 17 years, 27.2 ± 4.9 kg/m^2^ BMI. The Japanese populations' (*n* = 240; 50% women) age was on average 51 ±12 years with a mean BMI of 23 ± 2.9 kg/m^2^.

**Table 3:** Characteristics of Dutch, Portuguese and Japanese population.

	**The Netherlands *N* = 2,078**	**Portugal *N* = 3,792**	**Japan *N* = 240**
Gender			
Male [*n* (%)]	1,043 (50)	1,789 (47)	120 (50)
Female [*n* (%)]	1,035 (50)	2,003 (53)	120 (50)
Age (years) (mean±SD) (min-max)	50 ± 19 (19–80)	48 ± 17 (19–84)	51 ± 12 (31–76)
Bodyweight (kg) (mean±SD)	81 ± 16^a^	74 ± 15	60 ± 12
Height (cm) (mean±SD)	176 ± 10^b^	165 ± 10	161 ± 9
BMI (kg/m^2^) (mean±SD)	26.3 ±5.1^b^	27.2 ± 4.9	23.0 ± 2.9

a *Missing n = 3*.

b *Not measured n = 516*.

The average current consumption of seaweed was higher in Japan (9.7 g/day) than in the Netherlands and Portugal (on average 0 g/day in the total population in both countries) (Table 4). Seaweed consumption among consumers only in the Netherlands (*n* = 4) was approximately 15 g/day, which was higher than the average consumption in Japan among consumers only. The average current consumption of regular bacon (2.7 g/day) and regular lettuce (1.2 g/day) was higher among the Dutch population compared with the Portuguese population with (0.4 g/day) and (0.2 g/day), respectively. The consumption of pasta among the total population was similar in both countries with approximately 25 g/day.

**Table 4:** The daily average consumption of seaweed, bacon, lettuce in grams per day for Dutch, Portuguese, and Japanese adults' total study population and consumers only.

	**N**	**Mean**	**SD**	**P5**	**P25**	**P50**	**P75**	**P95**
**Netherlands**								
**Total population**								
Seaweed (g/day)	2078	0.0	1.1	0.0	0.0	0.0	0.0	0.0
**Consumers only**								
Seaweed (g/day)	4	15.2	13.6	4.6	4.6	15.0	25.0	25.0
**Foods in replacement scenario's**								
**Total population**								
Bacon (g/day)	2078	2.7	10.4	0.0	0.0	0.0	0.0	18.8
Lettuce (g/day)	2078	1.2	7.7	0.0	0.0	0.0	0.0	7.4
Pasta (g/day)	2078	25.8	67.9	0.0	0.0	0.0	29.4	120.1
**Consumers only**								
Bacon (g/day)	381	15.0	17.3	1.5	6.0	11.0	20.4	40.0
Lettuce (g/day)	144	15.2	21.8	1.7	4.6	10.0	20.0	49.0
Pasta (g/day)	716	73.3	86.9	8.7	23.9	62.5	99.3	189.6
**Portugal**								
**Total population**								
Seaweed (g/day)	3792	0.0	0.1	0.0	0.0	0.0	0.0	0.0
**Consumers only**								
Seaweed (g/day)	3	2.6	1.1	0.1	2.9	2.9	3.1	3.1
**Foods in replacement scenario's**								
**Total population**								
Bacon (g/day)	3792	0.4	4.4	0.0	0.0	0.0	0.0	1.1
Lettuce (g/day)	3792	0.2	2.7	0.0	0.0	0.0	0.0	0.0
Pasta (g/day)	3792	24.8	76.1	0.0	0.0	0.0	35.2	109.3
**Consumers only**								
Bacon (g/day)	238	6.2	14.4	0.4	0.9	2.3	8.3	24.0
Lettuce (g/day)	142	7.4	8.9	1.9	4.0	6.0	8.0	23.5
Pasta (g/day)	1510	63.0	94.1	13.3	26.0	43.8	73.4	175.2
**Japan**								
**Total population**								
Seaweed (g/day)	240	9.7	8.7	1.1	4.1	8.2	11.9	17.4
Seaweed WW (g/day)	240	8.0	8.3	0.0	2.4	6.6	10.6	15.1
Seaweed DW (g/day)	240	1.7	1.4	0.1	0.8	1.3	2.4	3.5
**Consumers only**								
Seaweed (g/day)	240	9.7	8.7	1.1	4.1	8.3	11.9	25.5
Seaweed WW (g/day)	226	8.5	8.4	0.6	2.7	6.8	10.8	22.8
Seaweed DW (g/day)	238	1.7	1.4	0.2	0.8	1.3	2.4	4.5

*WW, wet weight; DW, dry weight*.

The intake of selected nutrients (iodine and sodium) and exposure to contaminants (arsenic, cadmium, lead, and mercury) in adults in the Netherlands (Table 5) and in Portugal (Table 6) are displayed for both the reference scenario (left) and the alternative (seaweed) scenario (right). In addition, for comparison, intake and exposure estimates of the selected elements for Japan, a country with a current high consumption of seaweed, is provided in Table 7. In the same tables, also the respective HBGV's and BDMLs are shown to evaluate how the potential shift due to the modeled intakes and exposures relate to guidance values in the countries and across scenarios. Color codes in these three tables identify the modeled intake levels that are, based on the current methods, without health concern (indicated in green), are with health concern because of too low or too high intake/exposure (indicated in red), or are on the verge of too high intakes (indicated in orange).

**Table 5:** The intake of selected nutrients (iodine and sodium) and exposure to contaminants (arsenic, cadmium, lead, and mercury) in adults in the Netherlands for the reference and alternative scenario^a^.

	**Reference scenario**							**Alternative scenario**					
**Netherlands**	**Mean**	**(95%CI)**	**P50**	**P75**	**P95**	**(95%CI)**		**Mean**	**(95%CI)**	**P50**	**P75**	**P95**	**(95%CI)**
Iodine (ug/day)	181	(177,186)	171	214	296	(285,301)		300	(289,310)	277	362	531	(500,560)
Sodium(mg/day)^b^	2500	(2460,256)	2399	2921	3887	(3741,4027)		2520	(2480,2581)	2419	2943	3911	(3767,4052)
i-Arsenic (μg/kg bw/day)	0.25	(0.25,0.26)	0.24	0.3	0.41	(0.40,0.42)		0.25	(0.25,0.26)	0.24	0.3	0.41	(0.40,0.42)
t-Arsenic (μg/kg bw/day)^c^	0.67	(0.63,0.72)	0.61	0.82	1.25	(1.13,1.40)		1.02	(0.95,1.09)	0.91	1.24	1.95	(1.78,2.18)
Cadmium (μg/kg bw/day)	0.22	(0.21,0.22)	0.21	0.25	0.33	(0.32,0.34)		0.22	(0.21,0.22)	0.21	0.25	0.34	(0.32,0.34)
Lead (μg/kg bw/day)	0.55	(0.54,0.57)	0.52	0.65	0.9^d^	(0.86,0.94)^d^		0.55	(0.54,0.57)	0.52	0.65^d^	0.9^d^	(0.86,0.94)^d^
Mercury (μg/kg bw/day)	0.1	(0.10,0.11)	0.09	0.13	0.2^e^	(0.18,0.21)^e^		0.1	(0.10,0.11)	0.09	0.13^e^	0.2^e^	(0.18,0.21)^e^

a *Color codes identify intake levels that are without health concern (indicated in green), are with health concern because of too low or too high intake/exposure (indicated in red), or are on the verge of too high intakes (indicated in orange)*.

b *Exceeding safe and adequate intake of 2000 mg/day (EFSA)*.

c *Exceeding BMDL of 0.3-8 ug/kg bw/day i-arsenic (BMDL01,EFSA)*.

d *At risk or exceeding BMDL of 0.63 (f) ug/kg bw/day lead, (BMDL10,EFSA)*.

e *At risk for exceeding HBGV of 0,19 ug/kg bw/day MeHg [(P),TWI,EFSA]*.

**Table 6:** The intake of selected nutrients (iodine and sodium) and exposure to contaminants (arsenic, cadmium, lead, and mercury) in adults in in Portugal for the reference scenario and the alternative scenario^a^.

	**Reference scenario**							**Alternative scenario**					
**Portugal**	**Mean**	**(95%CI)**	**P50**	**P75**	**P95**	**(95%CI)**		**Mean**	**(95%CI)**	**P50**	**P75**	**P95**	**(95%CI)**
Iodine (ug/day)	142	(140,146)	131	172	254	246,265		208	(202,214)	188	255	395	377,407
Sodium(mg/day)^b^	2360	2329,2404	2234	2792	3859	3749,3949		2370	2337,2414	2243	2802	3867	3756,3958
i-Arsenic (μg/kg bw/day)	0.26	(0.25,0.26)	0.24	0.31	0.43	(0.42,0.44)		0.26	(0.25,0.26)	0.24	0.31	0.43	(0.42,0.44)
t-Arsenic (μg/kg bw/day)^c^	1.48	(1.41,1.55)	1.29	1.81	2.97	(2.71,3.18)		1.67	(1.59,1.75)	1.48	2.06	3.35	(3.08,3.60)
Cadmium (μg/kg bw/day)	0.23	(0.22,0.23)	0.22	0.27	0.37	(0.35,0.39)		0.23	(0.22,0.23)	0.21	0.27	0.37	(0.36,0.39)
Lead (μg/kg bw/day)	0.41	(0.40,0.41)	0.39	0.48	0.67^d^	(0.65,0.69)^d^		0.41	(0.40,0.41)	0.39	0.48	0.67^d^	(0.65,0.69)^d^
Mercury (μg/kg bw/day)	0.14	(0.14,0.15)	0.13	0.18	0.29^e^	(0.26,0.31)^e^		0.14	(0.14,0.15)	0.13	0.18	0.29^e^	(0.26,0.31)^e^

a *Color codes identify intake levels that are without health concern (indicated in green), are with health concern because of too low or too high intake/exposure (indicated in red), or are on the verge of too high intakes (indicated in orange)*.

b *Exceeding safe and adequate intake of 2000 mg/day (EFSA)*.

c *Exceeding BMDL of 0.3-8 ug/kg bw/day i-arsenic , (BMDL01,EFSA)*.

d *At risk or exceeding BMDL of 0.63 (f) ug/kg bw/day lead, (BMDL10,EFSA)*.

e *At risk for exceeding HBGV of 0,19 ug/kg bw/day MeHg [(P),TWI,EFSA]*.

**Table 7:** Outcome of the assessment for the intake of selected nutrients (iodine and sodium) and exposure to contaminants (arsenic, cadmium, lead, and mercury) for Japan^a^.

	**Reference scenario**					
**Japan**	**Mean**	**(95%CI)**	**P50**	**P75**	**P95**	**(95%CI)**
Iodine (ug/day)^b^	2,320	(1,926, 2,825)	1,612	2,885	6,538	(5,155, 8238)
Sodium (mg/day)^c^^d^	4,470	(4,367, 4,594)	4,370	5,048	6,222	(5,894, 6462)
i-Arsenic (μg/kg bw/day)	0.92	(0.88,0.94)	0.89	1.06	1.37	(1.29,1.43)
t-Arsenic (μg/kg bw/day)^e^	4.8	(4.62,5.04)	4.51	5.72	8.07	(7.51,8.64)
Cadmium (μg/kg bw/day)^f^	0.52	(0.50,0.54)	0.5	0.6	0.76	(0.73,0.80)
Lead (μg/kg bw/day)	1.01^f^	(0.98,1.04) ^f^	0.98 ^g^	1.16 ^g^	1.49^g^^h^	(1.43,1.56)^g^^h^
Mercury (μg/kg bw/day)^i^	0.32	(0.31,0.35)	0.3	0.38	0.52	(0.47,0.57)

a *Color codes identify intake levels that are without health concern (indicated in green), are with health concern because of too low or too high intake/exposure (indicated in red), or are on the verge of too high intakes (indicated in orange)*.

b *Exceeding the UL of 600 ug/day (EFSA)*.

c *Exceeding safe and adequate intake of 2000 mg/day (EFSA)*.

d *Including added salt*.

e *Exceeding BMDL of 0.3-8 ug/kg bw/day i-arsenic (BMDL01, EFSA 2014)*.

f *Exceeding HBGV of 0.36 ug/kg bw/day cadmium ((P)TWI)*.

g *Exceeding BMDL of of 1.5 ug/kg bw/day lead (BMDL01,EFSA)*.

h *At risk for exceeding BMDL of 0.63 ug/kg bw/day lead (BMDL10,EFSA)*.

i *Exceeding HBGV of 0.19 ug/kg bw/day mercury [(P)TWI,EFSA]*.

For iodine, mean (95%CI) and median intake levels in the Netherlands were, respectively, 181 (177, 186) μg/day and 171 μg/day. For the Portuguese population, the mean (95%CI) and median intake levels were 142 (140, 146) μg/day and 131 μg/day, respectively. The alternative scenario (with replacement of regular foods by 10% of seaweed foods) almost doubles the mean iodine intake in both countries, more pronounced in the Netherlands due to the higher consumption of bacon and lettuce compared with Portugal, resulting in 300 μg/day and 208 μg/day, respectively. The median iodine intake in the alternative scenario among the Portuguese population was 188 μg/day. In the alternative scenario the 95th percentile (95%CI) of iodine intake was on average 531 (500, 560) μg/day for the Netherlands and 395 (377,407) μg/day for Portugal. The alternative scenario resulted in neither of these two countries in exceeding the UL (600 μg/day). In contrast, in Japan, the mean intake of iodine (2,320 μg/day) with the current dietary habits exceeds by far the UL, 5% of the population exceed the UL more than 10 times.

For sodium, average intake levels for the reference and alternative scenario, in both the Netherlands (2,500 and 2,520 mg/day, respectively) and Portugal (2,360 and 2,370 mg/day, respectively) were comparable. There seems no incremental intake of sodium from the alternative scenario for both countries. In Japan, the mean intake of sodium (4,470 mg/day) (including added salt) was high and surpasses by far the safe and adequate intake level for the large majority of the population.

For t-arsenic, average exposure levels were, respectively, 0.67 μg/kg bw/day and 1.48 μg/kg bw/day for the Netherlands and Portugal in the reference scenario. The alternative scenario resulted in an increase of 50% in exposure compared with the reference scenario for both countries. The mean exposure to t-arsenic in the reference scenario falls within the EFSA BMDL01-range; thus the mean MoE (i.e., BMDL divided by the mean population exposure) is 1. Comparing mean t-arsenic exposure with the JECFA BMDL05 results in MoEs slightly above 1. In comparison, in Japan, exposure to t-arsenic was considerably higher (4.8 μg/kg bw/day), and falls within the range of EFSAs BMDL01 and above JECFAs BMDL05. In addition, it has to be noted that both these BMDLs are established for i-arsenic as it is the more toxic variant of arsenic.

For i-arsenic, the alternative scenario resulted in both the countries with no difference in exposure vs. the reference scenario, even though a modest increase was observed for t-arsenic. Compared with the reference dose derived by EFSA, the mean exposure to i-arsenic in both the reference and alternative scenarios were slightly below the range of the BMDL01 for both the Netherlands (0.25 μg/kg bw/day) and Portugal (0.26 μg/kg bw/day), and hence MoEs are above but close to 1. The 95th percentile of the population in both the Netherlands (0.41 μg/kg bw/day) and Portugal (0.43 μg/kg bw/day) were at an exposure level that is within the EFSA BMDL01 range. Comparing i-arsenic exposure with the BMDL0.5 derived by JECFA, MoEs were considerably higher for both countries (MoE of 12 using mean exposure in both countries). In comparison, in Japan, exposure to i-arsenic (0.92 μg/kg bw/day) was significantly higher and within the EFSA BMDL01 range, but below the JECFAs BMDL0.5.

For cadmium, exposure levels in both the Netherlands and Portugal were 0.22 and 0.23 μg/kg bw/day, respectively, in the reference scenario. The alternative scenario did not lead to an increase in the cadmium exposure in both countries, and consequently none of the evaluated HBGVs were surpassed, neither the lower (P)TWI of EFSA nor the higher (P)TMI of JECFA. In comparison, in Japan, cadmium exposure (0.52 μg/kg bw/day) was very high and surpassed the HBGV in nearly everyone.

For lead, mean exposure levels were 0.55 μg/kg bw/day for the Netherlands and 0.41 μg/kg bw/day for Portugal in the reference scenario, with lower MoEs for Netherlands, 1.1–2.7, than for Portugal, 1.5–3.7. The alternative scenario slightly decreased the lead exposure in both countries. For Japan, lead exposures were higher (1.01 μg/kg bw/day) compared with the Netherlands and Portugal, and on the verge of the BMDL for the general population or (for females) above the established BMDL.

For mercury, the speciation and the associated comparator HBGV is important. In the reference scenario, the average exposure to mercury was 0.10 μg/kg bw/day for the Netherlands and 0.14 μg/kg bw/day for Portugal. If total exposure to mercury is compared with the HBGV for t-mercury (0.57 μg/kg bw/day), the HBGV is not surpassed, neither in the Netherlands nor in Portugal. However, the 5% with the highest exposure levels to mercury exceed the HBGV, if, in a precautionary mode, all mercury is considered to be methyl-mercury in the reference scenarios. The alternative scenario did not lead to higher exposure levels of mercury. Again, in nearly everyone in Japan exposure to mercury exceeds the HBGV for methyl-mercury, but not t-mercury.

Supplementary Table 1 gives an overview of the top food products, which contribute the most to the exposure to chemical contaminants for each country; this reflects the existing differences in food consumption (diet) between the countries. Seaweed bacon, seaweed pasta, and fish were the primary contributors to t-arsenic exposure in the alternative scenario in the Netherlands; in Portugal, seaweed pasta and fish were in the top four contributing foods to t-arsenic exposure. In addition, the top five contribution foods for t-arsenic in the reference scenario in Japan included rice and seaweeds, whereas for i-arsenic the top five products did not include seaweed in all the three countries. These results indicate that the consumption of seaweed highly influences the exposure to t-arsenic. No significant differences were noticeable between the scenarios for the other heavy metals.

Finally, Supplementary Table 2 provides an overview of the percentage (and lower and upper bound) of the adult population in the Netherlands and Portugal below the established HBGV's and BMDLs by EFSA and JECFA based on current estimations (in both the reference and the alternative scenario). For t-arsenic, >95% of the Dutch and Portuguese population exceeds the lower limit of the BMDL01 of 0.3–8 μg/kg bw/day for 1% increased risk of cancer in both the reference and alternative scenario; however the range between the lower and upper limit was very wide. In addition, in the Netherlands around 28% of the population exceeds the BMDL10 of 0.63 μg/kg bw/day for lead for 10% increase in incidence for chronic kidney disease in both the reference and alternative scenario.

## Discussion

In this study, we explored the impact of an increased seaweed consumption on nutrient intake (iodine and sodium) and exposure to contaminants (t-arsenic, i-arsenic, cadmium, lead, and mercury) among Dutch and Portuguese adults. Results from this work revealed that the modeled increased seaweed consumption (as assessed by the 10% replacement with seaweed products) has no consequences on human health in terms of intake for sodium, and exposure to cadmium, lead, and mercury, and the associated (absence of) adverse health aspects. The intake of iodine and exposure to arsenic, both are certainly higher in the alternative seaweed scenario compared with the reference scenario. Compared with Japan, the nutrient intake and exposure to contaminants were lower in the Netherlands and Portugal.

In this first tier risk-benefit analysis, the alternative seaweed scenario was based on the current food intake derived from the Dutch and Portuguese national food consumption surveys, in which seaweed intake is currently relatively low. The replacement of 10% consumption of lettuce, pasta, and bacon with seaweed products is considered very optimistic for the short term. Even though consumption patterns do shift over time, it will take considerable time, if ever, before consumption of seaweed products takes over 10% of the market share of lettuce, pasta, and bacon. Lately, seaweed has raised increased public awareness in Europe because of several reasons: it rapidly grows in the sea and it has multifunctional use. Considering food security and global sustainability challenges, it can be expected that the consumption of seaweed increases in Europe due to its high nutritional value, combined with the expansion of the health-food industry (6). Therefore, it is important to monitor excessive intake of nutrients and elements from seaweed that could potentially harm human health, such as iodine or arsenic. In contrast to Dutch and Portuguese diets, seaweed foods are common foods in the Japanese diet. Compared to the current HBGVs and BMDLs, the Japanese population might be at risk because relatively high intakes of sodium and iodine were estimated. As expected, Japanese studies reported that higher sodium intake is identified as one of the major dietary health risks (62, 63). Small-scale studies have investigated iodine intake and thyroid dysfunction (64, 65); however, public health issues regarding iodine consumption in Japan have not been identified. More studies are needed to carefully evaluate health risks of higher intake of iodine and sodium in the Japanese population.

Generally, in risk-benefit assessment for food safety and nutrition, it is advised and practical to run the assessment in a tiered approach, as described in the EU BRAFO project (27), about a decade ago. This has been further developed in the RiskBenefit4EU project (31, 66). The risk-benefit question is addressed in this study by comparing intake and exposure levels in a reference and in an alternative scenario with established HBGVs and BMDLs. For the interpretation of study results, it should be noted that due to methodological limitations, i.e., not including all dietary sources of iodine or sodium and lacking data on contaminants, a fair comparison with the HBGVs or BMDLs is not possible as intakes might be underestimated. Considering the current low consumption of seaweed, our results provide no indications for further assessment with deeper tiers using health metrics such as disability/quality-adjusted life years (DALYs or QALYs).

On the basis of the intake and exposure assessment and compared with HBGVs, BMDLs and the reference scenario, it can be concluded that exchanging the reference scenario for the alternative scenario does not change the public health risks consequent to intake or exposure to sodium, cadmium, lead, and mercury. For iodine, even though the intake increases in the alternative scenario, the UL is not exceeded, not even by the 95th percentile consumers. Rather, a substitution of pasta, bacon, and lettuce by the seaweed alternatives will shift the intake distribution of the population to the right, thus potentially decreasing the risk of diseases associated with inadequate iodine intake. However, considering the methodological limitations, this intake might be underestimated. Whereas the study is restricted to adults, in some countries other population groups are at risk of too high or too low intakes of iodine. The present work did not include children who have a relatively high intake of milk or young girls who may consume less bread; milk and bread are major sources of iodine and add substantially to the dietary intake of iodine in the Netherlands and Portugal (67). Likewise, in case of high consumption of food supplements or iodized salt such may lead to concomitant high intakes of iodine (68).

For i-arsenic, the exposure in the reference scenario is for 25% of both the Dutch and Portuguese population (Supplementary Table 2) already within the range of the BMDLs derived by EFSA and might be considered as a health concern with respect to an increase in incidence of lung (BMDL0.5) and bladder cancer (BMDL01) (49, 69). Exposure to t-arsenic considerably increased in the alternative scenario compared with the reference scenario because *S. latissima* contains mostly organic arsenic. In a study by Brandon et al. it was mentioned that seaweed can have a significant contribution to the dietary exposure of i-arsenic (70). This is in agreement with results found in a study by Duinkers et al. in which they showed the different heavy metal content of different seaweed species collected in Norway (52). In general, >90% of t-arsenic in kelp consists of organic arsenic, primarily of arsenosugars which are known to have very low toxicity (52). It has to be noted that some seaweed species, such as the Hijiki seaweed which is frequently consumed in Japan, can contain higher amounts of i-arsenic (71). Subsequently, the substitution of another seaweed specie into current analysis might lead to different outcomes, which also applies for the other contaminants as levels may vary within species. Unfortunately, i-arsenic content was not specifically measured in the seaweed investigated in SEAFOODTOMORROW. Therefore interpretation of the impact of increased seaweed consumption on i-arsenic should be drawn with caution, as exposure levels are already at a level of health concern.

In our analyses for the Netherlands and Portugal, the exposure to chemical contaminants remains at safe levels with reference to established HBGV or reference points (cadmium, lead, and mercury), or they remain too high vs. the BMDLs (arsenic). The sodium intake remains too high, even when added salt was not included. The increase in iodine intake has a beneficial impact, moving a proportion of the population with intakes below adequate intake to a higher intake, although this might be underestimated. In case of t-arsenic and i-arsenic and risk of cancer, results should be interpreted with care. Any increase in exposure to i-arsenic increases the risk of cancer and concurrently, EFSA recommends a reduction in exposure to i-arsenic in general (50).

This then translates into the conclusion that there are no risk assessment objections to exchange the reference diet with a diet consisting of 10% seaweed in pasta, bacon, and lettuce from the perspective of exposure to iodine, sodium, cadmium, lead, and mercury. For arsenic and when compared to the BMDL for t-arsenic, the exposure is above the HBGV in all scenarios, and seems to increase with higher seaweed consumption, thereby increasing the risk further. Hence, for now it is not relevant to perform higher tiers of risk-benefit assessment. Increase of seaweed in the diet according to alternative scenario may lead to small increases in risks and small benefits which are surrounded by uncertainties. Currently, the presence of seaweed in the diet is nowhere near the amount in the alternative scenario. So both risks and benefits would be small and the net impact in terms of public health close to zero. However, if seaweed intake would increase by a substantial amount, it may be wise to investigate if risks can be somehow reduced or if there is a trade-off with benefits.

A major strength of this study is that it provides new evidence on the potential impact on human health, of the consumption of seaweed in the Netherlands and Portugal. For interpretation of study results there are some limitations noted. There are several limitations to the occurrence data used for the exposure assessments and nutrient intake which may have influenced our study results. Firstly, the amount of product specific data is very limited. FoodEx2 (revision 2) classification system has seven different hierarchy levels (36), whereas a higher hierarchy level indicates more specific description. However, foods that were expected to contain high levels of a certain contaminant were included and therefore important dietary sources are covered. Secondly, the occurrence data are derived from EFSA articles published between 2009 and 2014, suggesting the data are relatively old and perhaps not representative for the actual exposure levels derived from food consumption data. Ideally, occurrence data collection and food consumption measurements take place simultaneously. Thirdly, the occurrence data from the EFSA articles are based on mean occurrence levels measured across all European countries. Thus, the amount of country-specific (Portugal and the Netherlands) data used is very limited as it, only covered a portion of all the foods consumed in the Portuguese and Dutch surveys. Lastly, for Japan, a non-European country, occurrence data were based on the EFSA articles as no country-specific data were provided for the heavy metals. Foods only consumed in Japan were matched to the FoodEx2 codes of comparable European foods, implicating that the occurrence data used for Japan are merely an indication of the exposure levels. Although, the accuracy of the occurrence data used in this study can be questioned, it does give some indication on the exposure levels of the investigated heavy metals, indicating which heavy metal(s) might be of health concern for European populations. The data also give an indication on the top food contributors that cause high dietary exposure to the heavy metals analyzed. These results might trigger follow-up studies with more accurate occurrence data. Considering the assessment of nutrient intake, different sources were used to obtain information on food composition for Portugal. Neither the Portuguese food composition table nor country-specific EFSA informations or the WHO publication on iodine covered all foods consumed. In this work, we covered all foods that we expected to contain relevant levels of the considered substances. Missing concentration data were substituted with Dutch concentrations on iodine. Therefore values for the Portuguese intake could differ. Since, in the Dutch food composition table it is assumed that bread was manufactured with iodized salt, concentrations might slightly differ. The usage of iodized salt is voluntary in Portugal and there are currently no regulations regarding the concentration of iodine or for iodine in iodized salt. The estimated iodine intake derived from bread might therefore be overestimated in the Portuguese diet. However, since the use of supplements, recommended for pregnant women in Portugal, and added iodized salt at home is not included in the current work, iodine intake might be underestimated. In general, for the interpretation of the results, for the Netherlands, Portugal, and Japan as well, it should be noted that estimation of iodine intake is rather complex. Besides the large variation of iodine concentrations in foods and the uncertain use of iodized salt, iodine intake estimation in diets is often an underestimation (72). The estimates that are compared with the HBGV's should therefore be interpreted with caution. Iodine intake is usually estimated from several sources [supplements, household salt (iodized salt) and (a part of) iodized salt used in manufactured foods (processed foods)], but not included in this work. If the population is at risk of high iodine intake due to the substitution of seafood products, ideally further research should be undertaken to assess iodine intakes, preferably by a 24-h urinary study.

In case more foods in the diet are replaced by seaweed-derived products this will require a new or adapted risk-benefit assessment at a higher tier. The use of seaweed for the production of foods may trigger the Novel Foods Regulation (73) and producers may need to build a complete dossier to support an application.

To conclude, the modeled increased seaweed consumption (as assessed by the 10% replacement with seaweed pasta, bacon and lettuce) has no consequences in terms of intake for sodium and exposure to cadmium, lead and mercury, and consequently the associated (absence of) adverse health aspects for the Netherlands and Portugal. Intake of iodine and exposure to arsenic are certainly higher due to the modeled seaweed consumption. If seaweed consumption increases close to the 10% substitution, the public health consequences thereof may trigger further research.

## Data Availability Statement

The datasets used during the current study are available from the corresponding author on reasonable request. Requests to access these datasets should be directed to Reina Elisabeth Vellinga, reina.vellinga@rivm.nl.

## Ethics Statement

Ethical review and approval was not required for the study on human participants in accordance with the local legislation and institutional requirements. Written informed consent for participation was not required for this study in accordance with the national legislation and the institutional requirements.

## Author Contributions

JH, ET, MS, and RV contributed to the conception, design, data collection, statistical analyses, data interpretation, manuscript drafting, approval of the final version of the manuscript, and agreed for all aspects of the work. BT was responsible for the data collection on seaweed. KG was responsible for chemical analysis. MS and DT supplied intake data. HV, GR-H, and LJ contributed to the data interpretation, manuscript drafting, approval of the final version of the manuscript, and agreed for all aspects of the work. All authors contributed to the article and approved the submitted version.

## Funding

This project had received funding from the European Union's Horizon 2020 Research and Innovation Program under Grant Agreement No. 773400 (SEAFOODTOMORROW).

## Author Disclaimer

This output reflects the views of the author(s), and the European Union cannot be held responsible for any use which may be made of the information contained therein.

## Conflict of Interest

The authors declare that the research was conducted in the absence of any commercial or financial relationships that could be construed as a potential conflict of interest.

## Publisher's Note

All claims expressed in this article are solely those of the authors and do not necessarily represent those of their affiliated organizations, or those of the publisher, the editors and the reviewers. Any product that may be evaluated in this article, or claim that may be made by its manufacturer, is not guaranteed or endorsed by the publisher.

## Abbreviations

(P)TMI: (Provisional) Tolerable Monthly Intake
(P)TWI: (Provisional) Tolerable Weekly Intake
AI: Adequate Intake
BMD: bench mark dose
BMDL: benchmark dose lower confidence limit
BMDL01: benchmark dose lower confidence limit for a 1 % increased incidence
BMDL0. 5: benchmark dose lower confidence limit for a 0.5 % increased incidence
CI: confidence interval
DALY / QALY: Disability adjusted life year/Quality adjusted life year
NEVO: Dutch food composition table
DNFCS: Dutch National Food Consumption Survey
EFSA: European Food Safety Authority
EU: European Union
JECFA: FAO/WHO Joint Expert Committee on Food Additives
HBGV: health based guidance value
i-arsenic: inorganic-arsenic
LOD: limit of detection
LOQ: limit of quantification
LNN: Logistic Normal Normal
MoE: margin of exposure
IAN-AF: National Food and Physical Activity Survey
NVWA: Dutch Food and Consumer Product Safety Authority
TSH: thyroid-stimulating hormone
UL: Tolerable Upper Intake Level
t-arsenic: total-arsenic (organic and inorganic arsenic)
WFSR: Wageningen Food Safety Research
WHO: World Health Organization.
