# Supplementary material for: Increasing Seaweed Consumption in the Netherlands and Portugal and the Consequences for the Intake of Iodine, Sodium, and Exposure to Chemical Contaminants: A Risk-Benefit Study
Source: Front Nutr. 2022 Jan 6;8:792923. doi: 10.3389/fnut.2021.792923 (PMC8770327; doi:10.3389/fnut.2021.792923)
Supplement: Supplementary file 1 [file Data_Sheet_1.docx]

Supplementary Material

# Supplementary Tables

**Supplemental Table 1. Overview of the top 5 food products, which contribute the most to the exposure to chemical contaminants for the Netherlands, Portugal and Japan.**

|  | **Total Arsenic** | **Inorganic Arsenic** | **Cadmium** | **Lead** | **Total Mercury** |
| --- | --- | --- | --- | --- | --- |
| **Reference scenario the Netherlands** | 1. Cod  2. Prawn, giant tiger  3. Fish (meat)  4. Shrimps and prawns  5. Salmons | 1. Tea infusions  2. Tap water  3. Wheat bread and rolls  4. Beer  5. Rice | 1. Potatoes and fries  2. Wheat bread and rolls  3. Carrots  4. Firm/semi-hard cheese (gouda and edam type) 5. Spinaches | 1. Tap water  2. Single meal replacement for weight reduction  3. Tea infusions  4. Beer  5. Coffee | 1. Tea infusions  2. Coffee  3. Canned tunas and similar  4. Cod  5. Fish (meat) |
| **Seaweed scenario, the Netherlands** | 1. Seaweedbacon  2. Seaweedpasta  3. Cod  4. Prawn, giant tiger  5. Fish (meat) | 1. Tea infusions  2. Tap water  3. Wheat bread and rolls  4. Beer  5. Rice | 1. Potatoes and fries  2. Wheat bread and rolls  3. Carrots  4. Firm/semi-hard cheese (gouda and edam type) 5. Spinaches | 1. Tap water  2. Single meal replacement for weight reduction  3. Tea infusions  4. Beer  5. Coffee | 1. Tea infusions  2. Coffee  3. Canned tunas and similar  4. Cod  5. Fish (meat) |
|  | | | | | |
| **Reference scenario, Portugal** | 1. Salted cod  2. Hakes  3. Octopus, common  4. Canned tunas and similar  5. Shrimps, common | 1. Still natural mineral water  2. Rice  3. Wheat bread and rolls  4. Tap water  5. Cow milk, semi skimmed | 1. Potatoes and fries  2. Wheat bread and rolls  3. Squids  4. Carrots  5. Chicken fresh meat | 1. Tap water  2. Potatoes and fries  3. Wine  4. Wheat bread and rolls  5. Tea infusions | 1. Salted cod  2. Canned tunas and similar  3. Hakes  4. Fish (meat)  5. Tuna |
| **Seaweed scenario, Portugal** | 1. Salted cod  2. Hakes  3. Octopus, common  4. Seaweedpasta  5. Canned tunas and similar | 1. Still natural mineral water  2. Rice  3. Wheat bread and rolls  4. Tap water  5. Cow milk, semi skimmed | 1. Potatoes and fries  2. Wheat bread and rolls  3. Squids  4. Carrots  5. Chicken fresh meat | 1. Tap water  2. Potatoes and fries  3. Wine  4. Wheat bread and rolls  5. Tea infusions | 1. Salted cod  2. Canned tunas and similar  3. Hakes  4. Fish (meat)  5. Tuna |
|  | | | | | |
| **Reference scenario, Japan** | 1. Rice  2. Algae, "Kombu"  3. Algae, "Hijiki"  4. Algae, purple laver  5. Algae, "Wakame" | 1. Rice  2. Tea, infusions  3. Tap water  4. Mushrooms  5. Coffee | 1. Rice  2. Japanese radishes, Daikon  3. Mollusks  4. Squids, raw  5. Carrot, regular (European type) | 1. Rice  2. Tea, infusions  3. Tap water  4. Beer  5. Soup stock | 1. Tea, infusions  2. Rice  3. Surimi products, "Yaki-chikuwa" (baked tubular kamaboko)  4. Tuna  5. Fish (meat) |

Supplemental Table 2. Overview of the percentage (and lower and upper bound) of the adult population in the Netherlands and Portugal below the established HBGV’s and BMDLs by EFSA and JECFA.

|  | | | Netherlands | | Portugal | |
| --- | --- | --- | --- | --- | --- | --- |
|  | HBGV/BMDL |  | % [LB-UB] Reference scenario | % [LB-UB] Seaweed scenario | % [LB-UB] Reference scenario | % [LB-UB] Seaweed scenario |
| Inorganic Arsenic | BMDL01:  0.3-8 μg/kg b.w. per day [56] |  | 75 [73-77] | 75 [73-77] | 73 [71-75] | 73 [71-75] |
|  | BMDL0.5:  3.0 μg/kg b.w. per day [47] |  | 100 [100-100] | 100 [100-100] | 100 [100-100] | 100 [100-100] |
| Cadmium | (p)TWI: 2.5 ug/kg bw per week [23] |  | 97 [96-98] | 97 [96-98] | 94 [92-96] | 94 [92-96] |
| Lead | BMDL01: 1.5 μg/kg bw per day [58] |  | 100 [100-100] | 100 [100-100] | 100 [100-100] | 100 [100-100] |
|  | BMDL10: 0.63 μg/kg bw per day [58] |  | 72 [68-75] | 72 [68-75] | 93 [92-94] | 93 [92-94] |
| Total Mercury | (p)TWI: 4 ug/kg bw per week [59] |  | 100 [100-100] | 100 [100-100] | 100 [100-100] | 100 [100-100] |
